# Interface Matters: The Stiffness Route to Stability of a Thermophilic Tetrameric Malate Dehydrogenase

**DOI:** 10.1371/journal.pone.0113895

**Published:** 2014-12-01

**Authors:** Maria Kalimeri, Eric Girard, Dominique Madern, Fabio Sterpone

**Affiliations:** 1 Laboratoire de Biochimie Théorique, Institut de Biologie Physico-Chimique, Centre National de la Recherche Scientifique, UPR9080, Univ. Paris Diderot, Sorbonne Paris Cité, Paris, France; 2 Univ. Grenoble Alpes, Institut de Biologie Structurale, Grenoble, France; 3 Centre National de la Recherche Scientifique, Institut de Biologie Structurale, Grenoble, France; 4 Commissariat à l'Energie Atomique et aux énergies alternatives, Institut de Biologie Structurale, Grenoble, France; National Institute for Medical Research, Medical Research Council, London, United Kingdom

## Abstract

In this work we investigate by computational means the behavior of two orthologous bacterial proteins, a mesophilic and a thermophilic tetrameric malate dehydrogenase (MalDH), at different temperatures. Namely, we quantify how protein mechanical rigidity at different length- and time-scales correlates to protein thermophilicity as commonly believed. In particular by using a clustering analysis strategy to explore the conformational space of the folded proteins, we show that at ambient conditions and at the molecular length-scale the thermophilic variant is indeed more rigid that the mesophilic one. This rigidification is the result of more efficient inter-domain interactions, the strength of which is further quantified via ad hoc free energy calculations. When considered isolated, the thermophilic domain is indeed more flexible than the respective mesophilic one. Upon oligomerization, the induced stiffening of the thermophilic protein propagates from the interface to the active site where the loop, controlling the access to the catalytic pocket, anchors down via an extended network of ion-pairs. On the contrary in the mesophilic tetramer the loop is highly mobile. Simulations at high temperature, could not re-activate the mobility of the loop in the thermophile. This finding opens questions on the similarities of the binding processes for these two homologues at their optimal working temperature and suggests for the thermophilic variant a possible cooperative role of cofactor/substrate.

## Introduction

Temperature is considered the main environmental factor that affected the amino acid composition of proteome during evolution [1]–[3]. Details of how temperature global changes influenced the capability of individual proteins to work optimally in different thermodynamic conditions are still debated. Proteins from extremophilic organisms, especially thermophiles, represent a natural model to investigate the issue and in particular the relationship between protein stability, flexibility and function [4], [5].

Thermophilic and hyperthermophilic proteins are indeed stable and functional at very high temperatures, up to the boiling point of water in some cases [6]–[8]. However, although folded in a native structure, they generally lack activity at ambient conditions [9]. Thus, assuming a direct correlation among function and flexibility, it was postulated that these proteins are intrinsically more rigid than their mesophilic homologues working at ambient temperature. The flexibility required for functionality is only recovered upon thermal excitation. This so called *corresponding states* principle [10] was first introduced by studying the lactate dehydrogenase (LDH) protein from organisms adapted to various thermal environments. In the seminal work by Somero [10], flexibility is meant, in a broad sense, as the capability of the protein to sample conformational states relevant for its activity.

The rigidity/stability relationship has found support for some homologues from experimental studies, e.g. using the hydrogen/deuterium exchange technique that probes proteins' soft-modes exposing amide groups to solvent [11], [12]. However, investigations on different homologous pairs as well as using techniques monitoring flexibilities at different length and time scales questioned the generality of the rigidity paradigm [13]–[17]. Computational studies also reported opposite views when characterizing the relative flexibilities of homologous proteins and their temperature dependencies [18]–[23].

Crystallographic studies on thermophilic proteins have proposed a correlation between the alleged protein rigidity and some structural motifs, i.e. the surplus of ion pairs (IP) and hydrogen bonds (HB), the presence of shorter loops and anchored -C and -N terminals as well as extended hydrophobic packing [6]. However, some of these factors might be the source of enhanced flexibility too, as it is shown in numerous computational studies reporting on the dynamics of HB/IP networking [19], [24] and hydrophobic contacts [25]. For a complete understanding of the molecular basis of thermal stability it is also important to consider the effect of oligomerization, and in particular the binding strength of the molecular interfaces, on the overall stability and flexibility of an oligomeric assembly as compared to those of the individual domains [6].

In the present work we tackle this problem by considering a pair of orthologous bacterial tetrameric malate dehydrogenase proteins (MalDH) from *Chlorobium vibrioforme* (Cv) and *Chloroflexus aurantiacus* (Ca) which are adapted to mesophilic and thermophilic environments, respectively. Both enzymes belong to the superfamily of malate and lactate dehydrogenase (LDH) which is made of three different groups [26]. Cv and Ca MalDH belong to the group of malate dehydrogenase which are the closest homologs of LDH (LDH-like group) [26]. Malate dehydrogenase (MalDH) catalyzes the reversible oxidation of malate to oxaloacetate using the NAD+ coenzyme whereas LDH converts lactate to pyruvate. In this family several crystal structures have been resolved for a large number of organisms living at various temperatures [27]–[31].

From the structural point of view, it was shown for Cv and Ca MalDH, in agreement with the general trend, that thermal stability is correlated to an increased number of salt bridges and hydrogen bonds as well as aromatic interactions across the domain interfaces. For the more thermostable Ca MalDH, a reduced flexibility was also forecasted on the basis of a proline and alanine surplus [28].

Some of us, recently resolved anew, with a high-resolution, the crystal structure of Ca MalDH along with well defined networks of structural water [29]. Herein, we use this structure to perform extended Molecular Dynamics simulations at two different temperatures, 300 K and 360 K. The dynamics of the thermophilic Ca MalDH is systematically compared to that generated by its mesophilic counterpart Cv MalDH as well as their isolated monomers.

Anticipating our main results, we find that oligomerization induces a very important rigidification of the protein matrix, a perturbation that is especially pronounced for the thermophile. This induced rigidity has a direct impact on the expected mechanism of cofactor and substrate binding [32], [33] and opens questions on the similarities of the binding processes in homologous proteins. Finally, we estimate and dissect the strength of the dimer binding interfaces, and individuate this as a key factor for the enhanced stability of the thermophile.

## Methods

### Systems description

**Figure 1:**
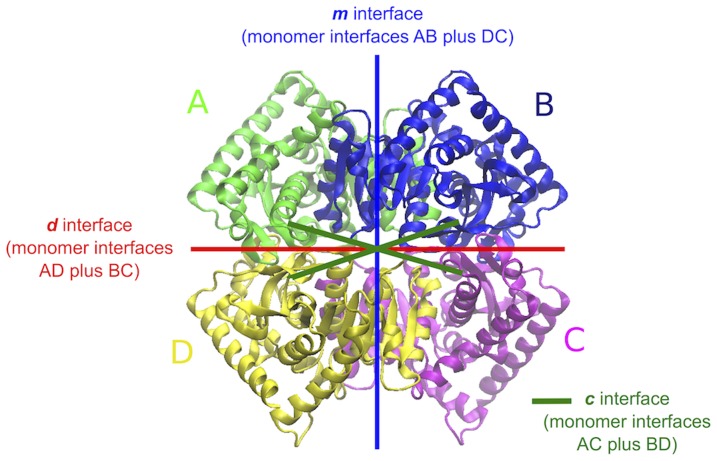
Structure of the MalDH tetramer. The above structure belongs to the thermophilic (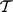) homologue but the chain and interface nomenclatures in the figure apply also for the mesophilic one (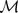). The tetramer is best described as a dimer of dimers. In the figure, the subunits A+B and C+D constitute each of the two dimers, which in turn interact with each other to form the tetramer. In order to study the interfacial interactions in a rather efficient way we have decomposed them into three different kinds comprising interfaces *m*, *d* and *c* the definition of which can be seen above.

We study two orthologous tetrameric malate dehydrogenases, a thermophilic extracted from the bacterium *Chloroflexus aurantiacus* (PDB code 4CL3 [29]) which is denoted from here on by 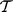 and a mesophilic from the bacterium *Chlorobium vibrioforme* (PDB code 1GV1 [28]) which is denoted by 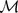. The two orthologues have a 74% sequence similarity (52.2% identity) with very similar structures; excluding the flexible loop at the top of the catalytic crevasse, the subunits are superimposed with an C*_α_*-*RMSD* of 1.0 Å. Fig. S1 shows an overlap of the two structures. The thermophilic species has 309 amino acids (a.a.) and the mesophilic one 310 a.a., per chain. The optimal growth temperatures of the organisms are 328 K (55°C) and 305 K (32°C) for 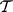 and 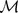, respectively [28]. Based on the characteristics of the different monomer-monomer interfaces and on the fact that the mesophilic homologue has been found to exist also in a dimeric form, the two tetramers are best described as a dimer of dimers [28]. In Fig. 1, the subunits A+B and C+D constitute each of the two dimers, which in turn interact with each other to form the tetramer. Previous studies on the LDH-like group of MalDH have shown that the minimal catalytic unit is made up by the dimer A+B or D+C [34], [35].

The selected proteins constitute the only pair of orthologues of the bacterial malate family for which crystallographic structures are available. An extra structure of a mesophilic bacterium exists (*Bacillus anthracis*, PDB code 3TL2) but the protein has not yet been characterized from the biochemical point of view. Crystallographic structures of thermophilic proteins from archaea exist but they belong to a separate phylogenetic group and cannot be used for a comparative study without including a bias due to organism evolution. The closest pair that can be considered for the sake of comparison belongs to the lactate family, namely the proteins from the mesophilic *Deinococcus radiodurans* (PDB code 2V6B) and thermophilic *Thermus thermophilus* (PDB codes 2V6M and 2V7P for the *apo* and *holo* states, respectively) [33].

### All-atom molecular dynamics simulations

All-atom Molecular Dynamics (MD) simulations were performed using the CHARMM22/CMAP force field for proteins [36] and the TIP3P-CHARMM model for water. The two systems were simulated in both their tetrameric and monomeric forms (i.e. simulation of an isolated domain). The tetrameric 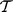 and 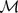 proteins were solvated respectively with 35422 and 38259 water molecules, while the two monomers with 7992 and 9009 water molecules. Counter-ions were added to neutralize the systems. All four systems were simulated both at 300 K and 360 K, for 200 ns for each temperature.

All simulations were performed using the NAMD software package [37]. The equations of motion were integrated using a time step of 2*fs*, with all bonds treated as flexible except for those involving hydrogen atoms which were kept rigid. Temperature and pressure were kept constant using the Langevin thermostat (with a dumping coefficient 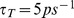) and barostat (with an oscillation period of 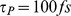), respectively. Electrostatics in a periodic simulation box was solved via the Ewald summation method and handled by the PME algorithm with a grid spacing of 1 Å. The production phases were preceded by 2*ns* of equilibration. The trajectories were dumped with a frequency of 4 ps.

#### Collective variables (CVs)

The radius of gyration was computed using the expression 
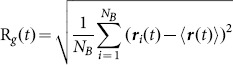
(1)where the summation is over all atoms, 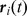 is the position of the *i-*th atom at time *t* and 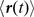 is the average position over all atoms at time *t*.

The Root Mean Square Displacement (*RMSD*) was computed via the following expression 
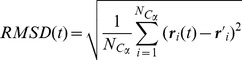
(2)where 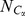 is the number of C*_α_* atoms in the chain, again 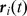 is the position of the *i-*th atom at time *t* and 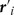 is its reference position in the crystal structure. Rigid body motions were removed by super-imposing the set of C*_α_* atoms of the protein configuration at time *t* on those of the crystal structure.

The number of native contacts 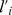 for a given side-chain heavy atom is the number of side-chain heavy atoms located within a cutoff distance of 5 Å in the crystallographic structure and being more than 3 residues apart in the sequence. Thus, the fraction of native contacts, referring to the whole chain, is defined as 
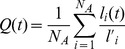
(3)where 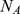 is the number of side-chain heavy atoms, having 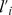 native contacts in the reference state and 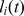 of them appearing also at time *t* (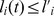).

The fraction of native torsion angles is given by 
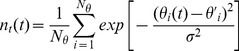
(4)where 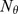 is the number of torsion angles 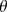, having values 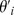 in the crystallographic structure and values 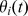 at time *t* and 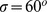. In our calculations the torsion angles along the sequence include both 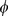 and 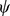 dihedrals.

Volumetric properties (i.e. volume per atom, compressibility and hydrophobic/hydrophilic surfaces) were calculated using the program *trjVoronoi* [38], [39].

The Root Mean Square Fluctuations (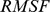) were computed via the following expression 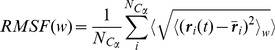
(5)where the two brackets denote a double time average. The inner average 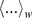 was calculated over a time window of 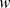 ps. To test the atomistic fluctuations at several time scales, the value of 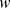 varied from 0.1 to 5 ns. The outer average 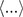 was calculated along the trajectory over all blocks of 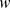 ps. The second averaging allowed to estimate the long time-scale variability of the 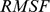. Finally, there was an averaging over all C*_α_* atoms of the sequence. For the 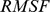 calculations of the tetrameric systems all 4 chains were considered.

#### Clustering

The clustering was done using the *leader* algorithm [40] and it is based on the pairwise root mean square deviations, as defined in Eq. 2 above, between different snapshots of the trajectory after removing rigid body motions and using a cutoff 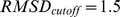 Å to separate different conformations. For the clustering, all heavy atoms were used. The results were verified to be robust by considering also the 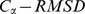 as well as different cutoff values (1 Å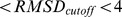 Å).

#### Diffusion

The diffusion coefficient for the proteins in the folded state was calculated for the collective variables 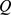 and 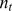. Generally speaking, given a collective variable 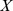, within the harmonic approximation [41], [42] the diffusion coefficient is given by 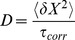, where 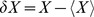 is the instantaneous fluctuation of the collective variable and 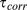 its correlation time, being defined as: 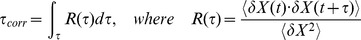
(6)


The autocorrelation in Eq. 6 decays exponentially after an initial short transient time. We used an exponential fit to estimate 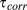. All the examined collective variables we study here have a fairly stationary behavior throughout the whole simulation length. Thus correlation functions were calculated for the entire trajectories excluding only, at the beginning of each, a stretch of 10 ns.

### Potential of mean force calculations

The potential of mean force (PMF) calculations were performed using the coarse-grained force field MARTINI v2.1 with polarizable water [43] and the simulation package Gromacs 4.6.3 [44]. The PMFs were calculated for the separation of only two bound domains each time. Namely we separately considered the separation of A from B, A from D and A from C for both 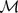 and 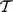. The starting conformations were as in the crystal structures. Each dimer (AB, AD and AC) was first solvated in a rectangular box of dimensions 75×80×260 Å and with the axis that connects the center of masses of the two domains being parallel to the z-axis. Our calculations followed the same protocol as in [45]. After an equilibration phase, the domains were forced apart. This procedure was necessary to generate the initial configuration for the umbrella sampling simulations [46]. The umbrella sampling was based on 60 to 70 windows depending on the system, separated one from the other by 0.5 Å. In each window the simulation run for 30 ns proceeded by a 10 ns equilibration phase. For the final profiles the Weighted Histogram Analysis Method (WHAM) was used [47] as implemented in Gromacs 4.6.3. The errors were estimated with bootstrap analysis.

## Results

### Conformational dynamics: insight into stability and function

#### Protein stability

We first point out that the two tetrameric systems are stable within the explored timescales and temperatures (see Fig. S2 of the SI). In fact, at ambient temperature the two systems fluctuate tightly around their crystallographic structures with a very low average 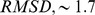 Å. At the higher temperature, the average value of the 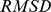 is slightly excited but remains lower than 3 Å for both systems. Things differ when the isolated monomers are considered. At ambient temperature, after a first drift that occurs for both systems within 40 to 80 ns, the 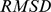
s show a steady behavior around the values 3.0 Å and 3.3 Å for 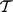 and 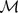, respectively. This conformational departure with respect to the X-ray structure is not surprising since it measures the lack of the packing/confinement of the tetrameric state. Interestingly, at 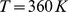 the mesophilic monomer shows signs of instability (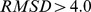 Å) localized at the curved helix stretch in the proximity of the active site (*α*1G-*α*2G). On the contrary the thermophilic monomer remains stable even at this high temperature.

Table 1 reports the radius of gyration, the volume per atom and the intrinsic compressibility data for all four systems and for the two temperatures. Within the error, the radius of gyration and the atomic volume are the same between the different orthologues, in either monomeric or tetrameric form. Thus, the enhanced thermal stability of 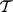 does not correlate to an improved atomic packing [8]. What we do note, however, is an important difference in the compressibility values. As noted for 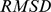, the monomers behave differently with respect to the tetramers. Indeed the intrinsic compressibility of the monomers, 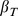, is higher than that of the tetramers as a signature of larger “breathing” modes and possibly a decreased stability [48]. Moreover, this difference is larger for the 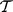 system and, as we will discuss widely later in the text, this indicates a strong, specific effect of the assembling in the tetrameric state of this species.

**Table 1:** Radius of gyration, volume and intrinsic compressibility.

		T = 300 K			T = 360 K		
System		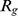 (Å)	V ( 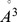 )	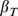 ( 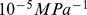 )	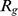 (Å)	V ( 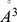 )	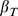 ( 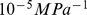 )
tetra	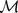	30.9±0.1	8.96 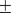 0.02	11.9 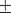 0.1	31.0 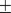 0.1	9.19 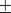 0.03	14.2 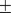 0.1
	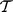	30.9 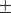 0.1	8.98 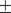 0.02	11.5 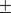 0.1	31.1 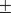 0.1	9.20 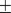 0.02	14.4 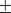 0.1
mono	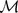	19.3 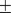 0.1	8.90 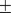 0.04	12.9 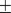 0.3	19.5 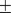 0.2	9.12 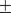 0.04	14.9 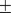 0.3
	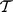	19.4 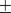 0.1	8.92 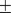 0.04	13.4 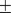 0.3	19.4 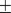 0.1	9.14 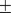 0.05	15.3 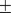 0.3

See methods for the calculation of the reported quantities. The errors correspond to standard deviation. The values of 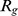
for chains in the tetramers are identical to those calculated in the isolated monomers.

#### Rigidity at atomistic length scales

We start investigating protein flexibility at the atomistic length scale. Neutron scattering experiments by M. Tehei et al. [49], probing the atomistic diffusion at small time scale (150 ps) of a hyperthermophilic malate (from *Methanococcus jannaschii*) as compared to a homologous mesophilic lactate dehydrogenase (from *Oryctolagus cunniculus*), suggested for the former a lower temperature dependence of atomic flexibilities. This behavior was also confirmed in silico by larger-scale simulations for the exact same pair of proteins [20].

**Figure 2:**
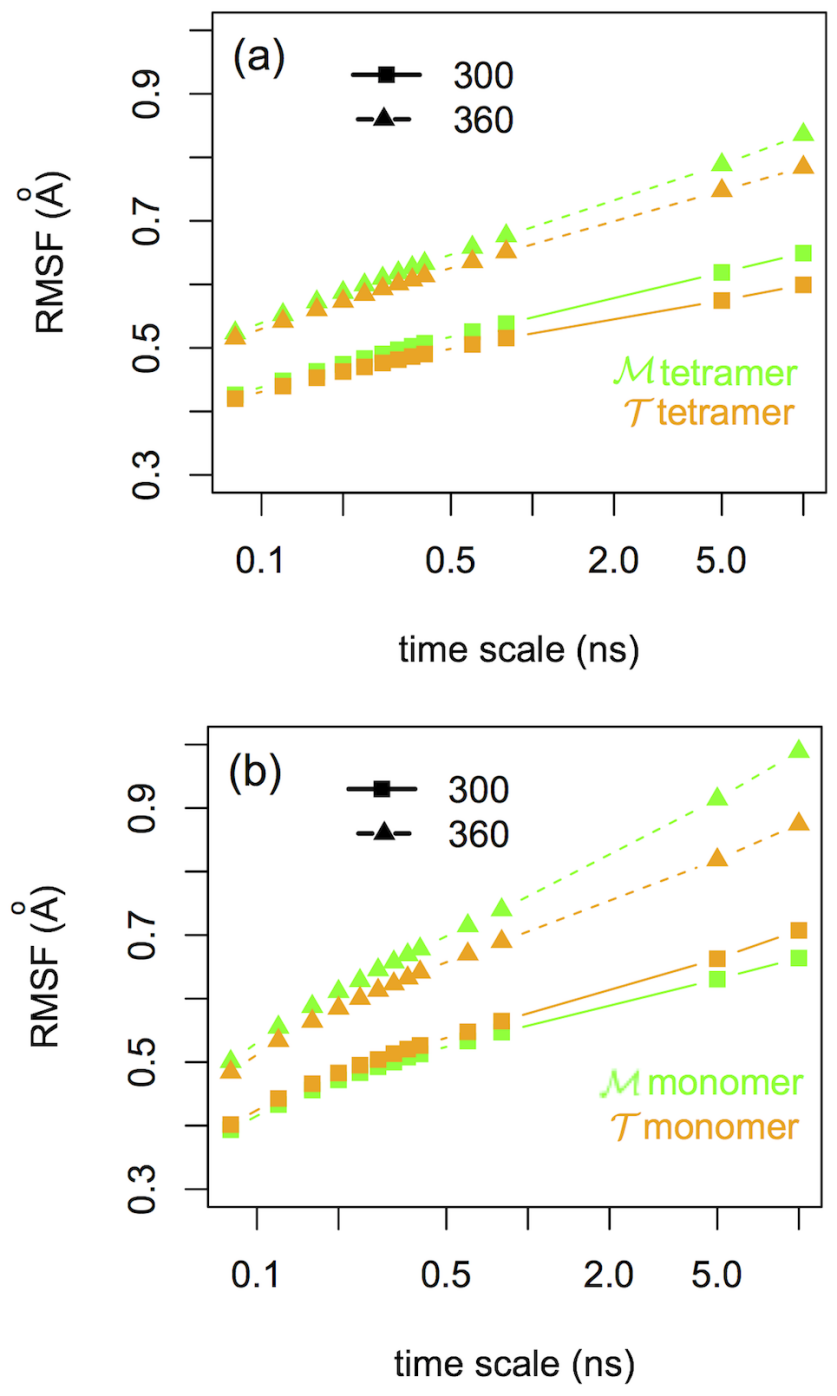
Flexibility at different time-scales. Average RMSF for 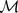 and 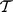 (a) tetramers and (b) monomers for both 300 K and 360 K.

Here, we use the root mean square fluctuations of C_α_ atoms or 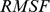 (see Methods), to examine to what extend our two 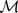 and 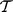 orthologues, that have a much larger sequence and structure identity than the pair mentioned above, comply to the previous observations. First, as opposed to what found in [20], the average 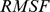 over all C_α_ atoms on short and long time-scales (up to 10 ns) shows that the tetrameric 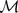 protein is more flexible than the tetrameric 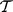 independently of the temperature (Fig. 2). When we look at the isolated monomers, at ambient temperature the relation reverses, and 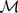 is now more rigid than 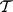. At the higher temperature of T = 360 K, the 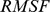 values of the 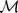 monomer become now larger than those for 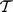, being this an extra indication of its kinetic instability. More importantly, by considering the shift due to the temperature increase, we also probe that our systems respond similarly (see Fig. S3 of the SI). In other words, the excitation of the atomistic fluctuations in the folded state (as sampled by our simulations) does not mirror the different thermal stabilities of our tetramers. A similar response to temperature increase has also been reported for other homologues [17].

#### Tetramer rigidity

**Figure 3:**
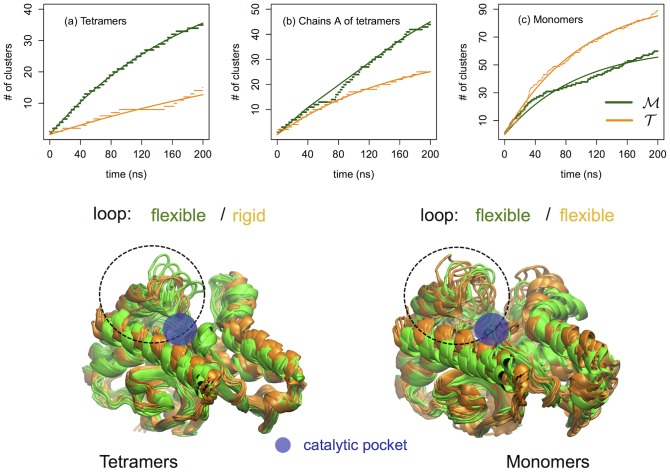
Conformational substates. Upper panel: Dotted lines correspond to the number of clusters at ambient temperature versus the simulation time for the (a) two 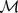 and 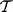 tetramers, (b) chains A of the 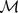 and 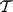 tetramers and (c) 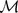 and 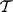 isolated monomers. Straight lines correspond to an exponential evolution fit of the form 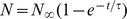. Lower panel: Thermophilic protein is in orange and mesophilic in green color. (Left) Cluster leaders for chain A of the tetramers' clustering and (right) cluster leaders of the monomers' clustering. Notice the anchoring of the loop at the active site of tetrameric 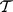 on the left with respect to the flexible loop of its isolated monomer on the right.

As a further step, we inquire into the rigidity of the proteins at a molecular scale by describing the conformational landscape explored by the systems at ambient temperature. The conformational states visited by the proteins are individuated using a clustering procedure (see Methods and [19]) based on the all-heavy-atom 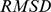. The total number of visited clusters versus time is extracted. The results are shown in the top panel of Fig. 3. When the monomers are isolated we notice that the 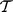 protein visits a larger number of conformational states than the 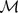 variant, see the right panel (c) of Fig. 3. Quite surprisingly the situation reverses when the simulations of the tetrameric systems are considered, with 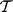 being significantly more rigid and exploring a smaller number of conformational states, see left panel (a) of Fig. 3. In order to quantify the effect of rigidification upon oligomerization, we have performed the clustering along the trajectories of the tetrameric systems but considering only one chain in the calculation. The results for chain A of 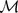 and 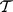 tetramers are given as an example in Fig. 3 (b). For the 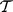 species the effect is quite important, indeed when in the tetrameric assembly the number of accessible states of the single chain is reduced by a factor of three. The estimated maximum number of clusters and the characteristic time of their saturation are obtained by fitting our data to an evolution function 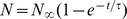 (see Table 2).

**Table 2:** Clustering: maximum number of clusters 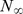 and their characteristic saturation time 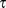.

		Clustering		Diffusion					
		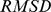		Fraction of native contacts 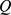			Fraction of native torsion angles 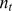		
System		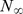	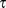 (ns)	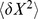	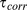 (ns)	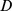	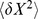	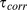 (ns)	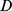
tetra	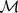	55	190	7	3.3	21	12	4.7	26
	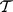	36	454	6	9.2	6	15	7.2	21
mono	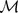	63	96	26	6.4	40	12	13.3	9
	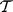	100	101	27	4.7	57	14	7.2	20

Diffusion: magnitude of CV fluctuations 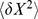, CV decorrelation time 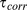 and the resulting diffusion coefficient 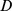.

Given that at ambient temperature the proteins are stable, the reported differences measure the relative flexibility of the proteins in their folded states. Comparing Figs. 3(b) and (c), it is clear that when the four monomers of each system come together the interfacial interactions between them have a rigidification effect on the protein matrix by reducing the number of accessible conformations. This effect is especially pronounced for the thermophilic variant 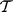.

The characteristic saturation times reported in Table 2 signal also the different kinetics of the proteins across the network of states. In a previous study of two homologous G-domain proteins [19], we found that the collective motion of the hyperthermophilic variant has a highly frictional character, i.e the native state is composed of multiple local minima separated by higher kinetic barriers that result in a slow internal diffusion with respect to that of its mesophilic counterpart. To quantify this diffusivity, the motion of the proteins with respect to a given collective variable (CV) was associated to a diffusion coefficient 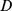. Within the harmonic approximation, 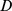 is given by the fluctuations of the CV divided by its characteristic decorrelation time, 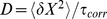. Interestingly, we herein agree with our previous findings. In fact for the tetramers the internal dynamics of 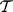 is about 20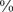 slower than that of 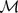. The data are shown in Table 2 for two CVs, namely the fraction of native contacts 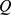 and the fraction of native torsion angles 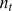 (see Methods). Just as for the G-domains, the fluctuations of the CVs are comparable between the two tetrameric systems but the decorrelation times are systematically larger for 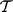 which reflects higher kinetic traps for this system. Again, the situation reverses for the case of the isolated monomers. The decorrelation time becomes now small and the diffusion coefficient larger for 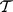. We note that two other tested variables, namely radius of gyration and 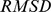 follow the same trend (data not shown).

#### Rigidity and ion-pair networks

The regions of the single domain that get mostly stiffened upon assembling in the tetrameric state are shown in the lower panel of Fig. 3 where the clustered conformations from the simulations at ambient temperature are represented. Conformations visited by chains A of the tetramers are shown on the left of the figure while the conformations visited by the isolated monomers are presented on the right. The mesophilic 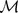 and the thermophilic 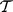 structures are overlapped and represented in green and orange, respectively. The largest effect is localized at the level of the loop at the entrance of the catalytic pocket. In fact, in its tetrameric form the 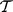 homologue maintains this loop in a closed state during all the simulation time, while in the isolated 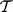 monomer the same loop is significantly more flexible. The respective region in 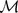 is equally flexible in either monomeric or tetrameric form. The different behavior of the loop in the 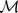 and the 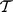 tetramers is observed in all the chains. This finding, as we will discuss later, might be important to dissect functional conformational changes occurring at the optimal working temperature of the thermophile. In fact, even if our tetrameric MalDHs, as other member of the LDH-like family, have never been crystallized in the presence of substrate analogues, the crystal structures of *apo* and *holo* LDH proteins [33] suggest critical conformational changes at level of this biding-site loop.

**Figure 4:**
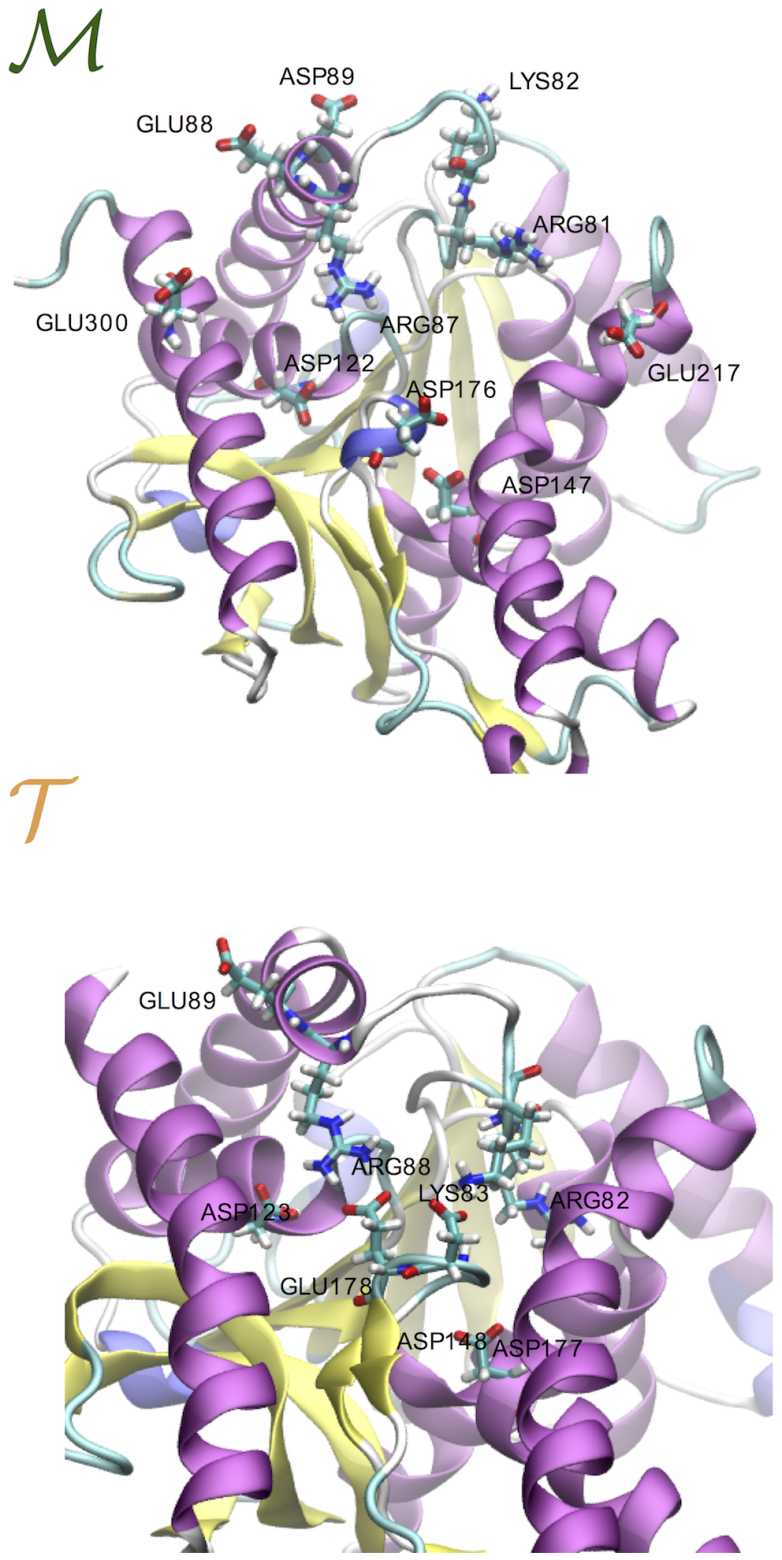
Salt-bridges at the external loop of the catalytic pocket. (Top) Mesophilic tetramer. The ion pairs (IPs) that form during the 200 ns simulation are Arg81-Glu217, Arg81-Asp147 and Arg81-Asp176, Lys82-Asp89 and Arg87-Asp176, Arg87-Asp122, Arg87-Glu300 and Arg87-Glu88. (Bottom) Thermophilic tetramer. The IPs that form during the 200 ns simulation are Arg82-Asp148, Arg82-Asp177 and Arg82-Glu178, Lys83-Asp177, Lys83-Glu178 and Lys83-Asp123 and Arg88-Glu178, Arg88-Asp123 and Arg88-Glu89.

At this point, the first question that arises is why in the 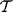 tetramer the loop is anchored down. The answer is found in the network of ionic interactions formed between this stretch of amino acids and the inner part of the catalytic pocket. First we note that in both homologues the loop hosts threes basic amino acids, namely Arg81, Lys82 and Arg87 in 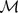 and Arg82, Lys83 and Arg88 in 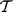 (see Fig. 4). As can be seen in Fig. 4 these residues can form several ion pairs with the acidic residues located inside the pocket. The fine differences between the sequences of the two proteins highlight two important features: first, residue Glu178 in 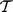 doesn't have an acidic analogue in 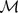 since at this position we find a hydrophobic amino acid (Ala177) and secondly, the salt-bridge Arg87-Glu300 in 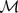 doesn't exist in 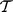 (upon sequence alignment of the two homologues, Glu300 is replaced by Ala301 in 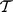 while at position 300 we find a positively charged arginine). These two factors are responsible for i) a reduced mobility of the loop in the 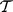 tetramer where the extra salt-bridge with Glu178 rigidifies the region and ii) for the increased flexibility of the loop in 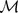 where the loop motion correlates to an alternating dynamics of ion-pairing of Arg87 with the partners Asp122 or Asp176 and Glu300. It is also worth noting that the arginine in position 81 (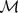) and 82 (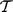) is conserved in all MalDHs, and its role during the enzymatic activity is well documented [26], [27]. In fact, this basic amino acid binds one of the carboxylates extremities of the substrate. Therefore, during the binding process, the ion-pairs formed by Arg81 (Arg82) must be replaced by the functional interactions with the substrate.

The second question that arises by looking at Fig. 3 is why for the thermophile 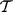 the loop is rigid in the tetramer and flexible in the monomer. At the molecular level this is due to a conformational funnel that constrains the residue Arg88 to closer distances with the partners Asp123, Glu178 and Asp177 (see Fig. 4). This locked state is caused by an acquired global rigidity of the protein matrix upon oligomerization. In fact, we verified that even by removing the motion of the loop, the number of conformational substates visited by the thermophilic tetramer is always smaller than for its mesophilic variant and the isolated monomer. The rigidity patterns are individuated by considering a drop in atomistic fluctuations upon oligomerization. The list of residues mostly affected by the stiffening are reported in the text of SI along with their molecular views (see Fig. S6). The striking result is that the rigidity pattern in 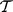 is more extended than the analogous in 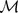 as well as more enriched in charged a.a. and long-lived IPs. This finding points out the important role of local electrostatic interactions in confining the conformational motion of the 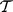 protein. A very similar difference in the a.a. composition is also found for the previously mentioned pair of mesophilic and thermophilic LDHs (2V6B and 2V7P) after structural alignment onto our MDHs and projection of the MDH rigidity patterns onto the LDH structures (see SI).

Concluding, we have verified that the packing of the interface causes a global rigidification of the 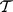 tetramer resulting in the anchoring of the binding site loop. The consequence of this locked state on the protein-substrate binding process will be addressed in the Discussion.

### Forces at the interfaces

#### Electrostatics and hydrophobicity

We now focus on the cause of the stiffening of the protein matrices by dissecting the energetics of the interdomain interfaces. In order to most effectively study the interfacial interactions we have decomposed the interfaces into three different types comprising *m*, *d* and *c* the definition of which can be seen in Fig. 1. Each one of them is the sum of two different monomer-monomer interfaces. For example the interface *m* is the sum of the interfaces between chains A and B as well as D and C.

The first three columns of Table 3 report the fraction of surface area of hydrophobic-hydrophobic, hydrophilic-hydrophilic and hydrophobic-hydrophilic (mixed) contacts along each of the three interfaces as estimated via Voronoi tessellation of the space [50] (Since the total interfacial area is not exactly the same for the two systems, to facilitate the comparison the surface has been normalized for each of the *m*, *d* and *c* interfaces, i.e. philic-philic, phobic-phobic and mixed add up to one. An additional table with the values in Å^2^ can be seen in SI, Table S1). The first observation is that *m* and *d* interfaces are favored hydrophobically for 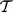 while they are favored hydrophilically for 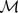. This is in line with certain structural facts; 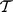 has, per chain, 10 more hydrophobic a.a. than 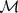 and in particular 1.5 times more along each of the *m* and *d* interfaces within a distance of 4.5 Å from hydrophobic a.a. of the opposite side. While both systems are slightly negatively charged the mesophilic has, per chain, 9 more charged a.a. than the thermophilic one. However, looking back in Table 3, the frustration (percentage of mixed surface) along the *m* interface is, for both systems, comparable with the sum of hydrophobic and hydrophilic surfaces. That roughly means that quantity doesn't matter there; it is rather the quality and specificity of interactions along this interface that result to a favorable free energy for the bound state of either system. In numbers, the average electrostatic energy stemming from inter-domain interactions is about 42% larger for 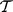 than that for 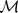 (see Table S2 in SI).

**Table 3:** Electrostatics and hydrophobicity at the interface.

		T = 300 K				
	System	S_hydrophobic_ (%)	S_hydrophilic_ (%)	S_mixed_ (%)	H-bonds	N*_IP_*
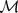	*m* interface	22 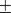 1	28 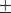 1	50 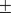 2	44 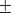 3	9.8 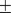 1.1
	*d* interface	27 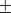 2	48 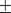 2	25 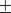 2	29 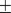 2	4.0 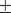 0.1
	*c* interface	13 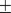 2	43 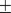 4	44 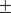 4	7 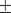 2	4.4 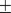 0.8
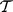	*m* interface	27 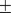 1	23 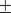 1	50 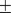 1	33 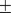 3	7.6 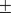 0.9
	*d* interface	35 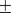 1	38 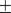 2	27 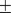 2	27 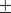 3	7.4 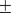 1.3
	*c* interface	7 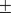 4	64 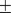 7	29 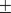 3	20 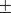 3	8.2 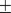 1.0
		T = 360 K				
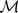	*m* interface	26 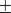 1	28 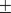 1	46 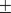 2	44 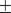 3	8.8 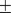 1.2
	*d* interface	31 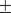 2	41 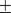 3	28 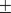 2	24 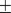 3	4.0 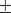 0.2
	*c* interface	16 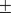 3	46 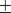 4	38 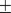 4	9 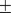 2	5 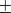 0.9
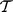	*m* interface	28 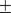 1	25 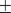 1	47 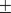 1	37 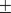 3	10.4 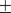 0.9
	*d* interface	34 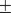 3	38 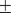 3	28 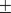 3	27 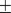 3	8.8 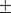 1.2
	*c* interface	10 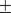 5	56 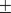 9	34 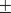 5	19 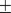 2	10 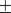 1.2

Errors correspond to standard deviation.

**Figure 5:**
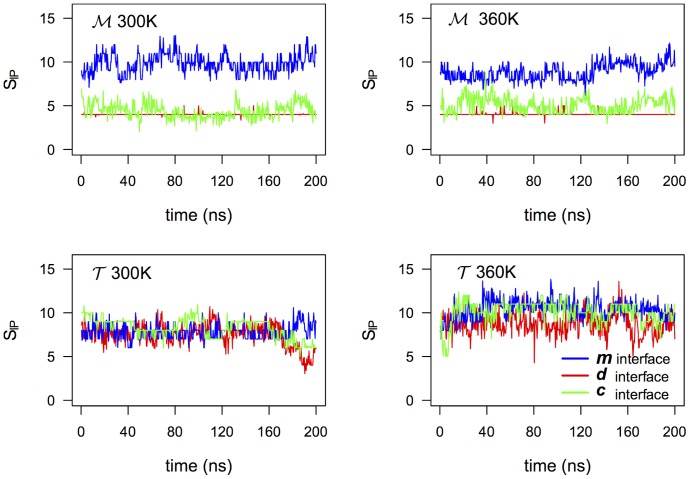
Ion-pairs on the three interfaces. Number of interdomain protein-protein ion-pairs on the 3 different interfaces of the tetramers at 300 K and 360 K. In Fig. S4 the HB timeline for both temperatures is also shown.

In this regard, the last two columns of Table 3, report the number of interdomain hydrogen bonds (HB) and ion-pairs (IP). In the thermophile, interfaces *d* and *c* have large numbers of either HBs or IPs, even if the number of charged a.a. is less than in 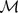. It is worth to note that along the three interfaces of the thermophilic variant we have a rather similar number of IPs and HBs, see Fig. 5 where the number of IPs is plotted as a function of time for the two systems at T = 300 K and 360 K. This uniform interfacial strain could contribute cooperatively to the global stiffening of the 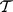 domains in the tetramer. We also stress that by increasing temperature, while the number of interfacial IPs tends to decrease in the 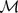 protein, it increases in 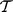 (see also Table 3 and Fig. 5). The higher connectivity in 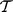 can be explained by the fact that the higher temperature facilitates small energy-barrier crossing events that favor new partnerships, while at the same time the two systems as a whole remain kinetically stable at the explored timescale. The possibility that IP large networking supports conformational changes across the interfaces during the enzymatic activity at high temperature is an appealing hypothesis to be investigated in future work.

#### Free energy of domain separation

**Figure 6:**
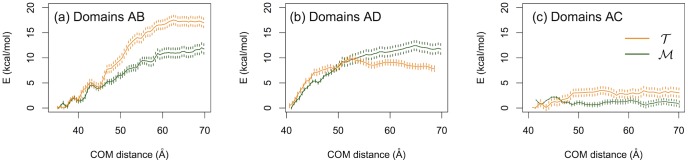
Potential of mean force profiles of domain separation. (a) Separation of domain A from B (see also Fig. 1), (b) Separation of domain A from D and (c) Separation of domain A from C.

We, additionally, estimated the strength of the interfacial matches by performing free energy calculations. Namely, we computed the work needed to separate the different domains of the two systems (see Methods). For computational reasons, we only considered the separation of two domains each time, and for our calculations we employed a coarse-grained model (MARTINI v2.1 with polarizable water [43]). The results are shown in Fig. 6. The leftmost panel (Fig. 6(a)) shows the potential of mean force to separate domain A from domain B. There is, clearly, a larger binding free energy for 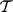 (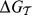 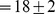 kcal/mol) as compared to 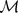 (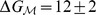
kcal/mol). On the other hand, the binding free energy for domains A and D are, within error, comparable for the two systems with a slightly larger value for the 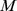 homologue, 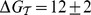 kcal/mol and 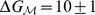 kcal/mol (Fig. 6(b)). Finally, the binding energy of domains AC are 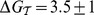 kcal/mol and 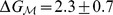 kcal/mol. Unfortunately, it is computationally very expensive to get a well converged potential of mean force for the dimer-dimer separation, that is the separation of dimer (A+B) from (C+D). However, our preliminary results indicate a larger binding free energy for 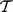 than that for 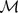 which is in line with previous experimental indications [28].

## Discussion

The melting temperatures of the mesophilic MalDH from *Chlorobium vibrioforme* and the thermophilic MalDH from *Chloroflexus aurantiacus* are 52.6°C (325.75 K) and 67.8°C (340.95 K), respectively [28]. Here, although the highest temperature in our simulations (360 K) is above both, we do not observe any signs of kinetic instability for the tetramers in the explored time scale. Interestingly though, at this temperature the isolated mesophilic monomer is less stable than its thermophilic homologue as revealed by higher conformational fluctuations (see Fig. S2 of the SI). Moreover, the region where this instability is localized is the curved helix (*α*1G-*α*2G), an important portion of which is part of the catalytic pocket. This finding indicates that the isolated domains set possibly a baseline in the tetramers' thermal resistance but extra stability is gained by domain-domain interactions [51].

Nevertheless, the important differences in the dynamics of the two homologues are revealed upon oligomerization. The main finding of our work is that in the tetrameric state the protein domains are systematically more rigid than in the isolated monomeric state and more importantly the rigidification process is very pronounced for the thermophilic variant. This was probed at both the atomistic and the molecular length-scales as well as considering volumetric properties. The 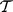 tetramer appears to be less compressible than 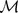, a relation that reverses when the monomers are considered isolated. In agreement to that, the internal motion of the 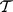 tetramer is slowed down with respect to 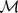 as effect of higher kinetic traps in the conformational landscape, a relation that also reverses for the isolated monomers. This picture is complimented with our cluster analysis of the explored conformational space; upon oligomerization both systems get stiffer yet the 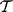 tetramer is confined in a much smaller conformational space than that of 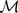.

By analyzing the X-ray structure, Dalhus and coworkers [28] forecasted a reduced flexibility for 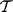 on the basis of the observed surplus of proline, however our finding points the attention to a more cooperative effect due to the interfacial packing. How the surplus of proline amino acids would contribute to enhance the domain rigidity upon oligomerization is an open question and relates to how interfacial packing transmits rigidity across the protein matrix. This will be the focus of a forthcoming work.

A structural comparison identified a few other factors as responsible for the increased thermal stability of 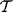, for example the increased number of alanine and aromatic residues on the *m* interface [28]. This finding in conjunction with our estimated gap between the domain binding free energies of 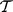 versus 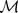 (Fig. 6(a)) reveals the importance of hydrophobic interactions along this interface [52].

For the other two types of interfaces, namely *d* and *c*, the thermophilic tetramer 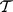, even if depleted in charged amino-acids with respect to the 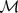 homologue, presents an higher number of both ion-pairs and hydrogen-bonds (see Table 3 and Figs. 5 and S4 in SI). However, for these interfaces, the free energy calculations do not mark any meaningful stability gaps between the two homologues. The role of ionic groups at the interfaces of Ca MalDH was investigated by single point mutations obtaining different results depending on the targeted amino acids [53], [54]. It was shown that when residue Glu25 that is located at the *c* interface was mutated to both a lysine and a glutamine the thermal stability at pH 7.5 is only slightly decreased, on the other hand when the Glu165, that belongs to the same network of ionic interactions (see Fig. S5 in SI), is mutated in a similar way, the thermal stability of the protein increases by ∼25 degrees without compromising the catalytic activity. Our simulations showed that at the *c* and *d* interfaces 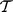 not only has a higher number of IPs and HBs but also a higher degree of connectivity. The charged residues in 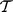 are placed along the interfaces in such a way so that they are topologically able to interact with multiple partners belonging to different domains. The patterns of this connectivity, absent in 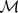, are represented in Fig. S5 and could play a role on the protein functionality by controlling long range motion and domain communication during the protein activity. Because of the extension of these ionic interactions, an adequate computational method and model should be used to obtain a more precise estimate of the ionic contribution to the protein stability [55].

The overall rigidity of the thermophilic protein in combination with local sequence specificities have an important consequence on the binding site dynamics. Namely, we refer to the external loop that upon formation of the enzyme-coenzyme-substrate ternary complex (MalDH/NAD/NADH) closes to act as a screening gate to the catalytic vacuole. Resent simulations of the dimeric MalDH from *Thermus thermophilus* with NAD showed that the loop, having started from an open conformation, closes during the simulation in order to bring key residues in contact with the co-substrate [30]. For the thermophilic malate under study, both the crystal structure and its 200-nanosecond dynamics are characterized by a constantly closed loop although the protein is coenzyme- and substrate- free. On the other hand, the respective loop in the mesophilic protein undergoes several openings and closings during the course of the simulation.

**Figure 7:**
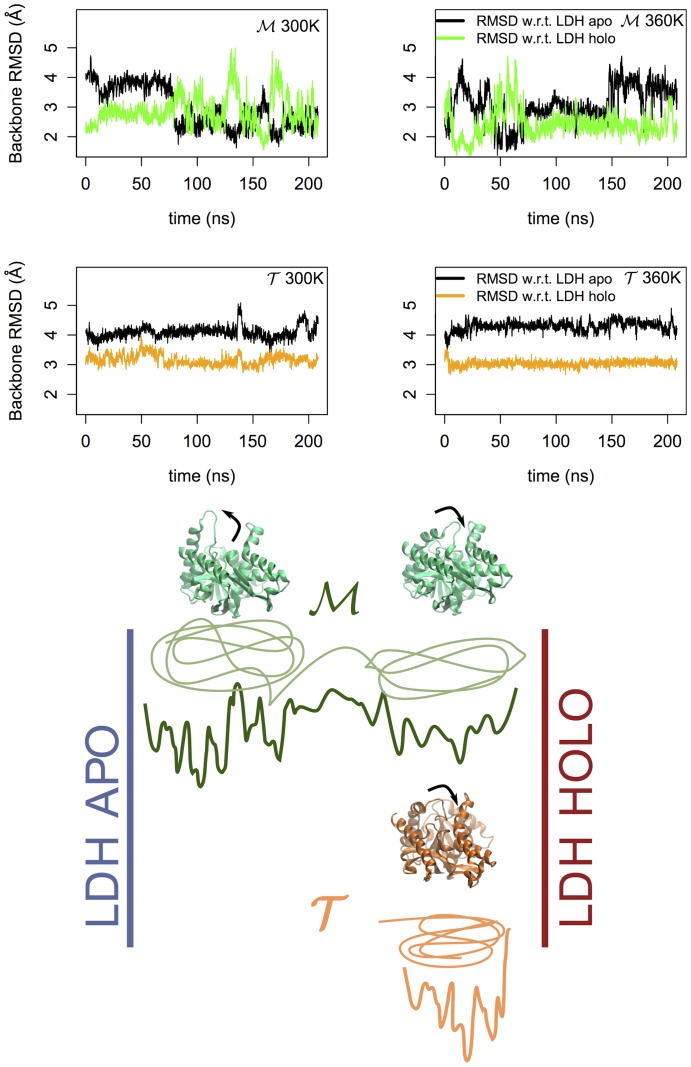
Catalytic pocket dynamics. In the upper part of the figure we report the RMSD computed using the backbone heavy atoms of the residues that form the catalytic pocket of 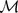 (upper graphs) and 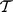 (bottom graphs) w.r.t. those of the *apo* and *holo* forms of lactate dehydrogenase at 300 K (left) and 360 K (right). In the lower part of the figure we present a pictorial representation of the conformational states accessible by the proteins when considered as function of the distance with respect to LDH *apo* and *holo* conformers. In green we sketched the two states visited by the 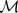 MalDH, one characterized by an open conformation of the binding site loop and the other associated to a closed state. The 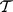 MalDH (orange) is instead tightly confined in a closed state.

To quantify this observation, we used as reference a third orthologous protein, a lactate dehydrogenase (LDH), whose crystal structure has been fully resolved in both holoenzyme and apoenzyme conformation [33]. For the simulated trajectories we calculated the 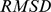 of the backbone atoms of the residues that form the catalytic pocket for 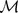 and 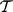 with respect to both the *apo* and *holo* form of LDH. The results can be seen in Fig. 7. While the mesophilic tetramer switches intermittently between conformations close to the LDH *apo* form and LDH *holo* form, the thermophilic tetramer remains rigidly around a conformation that mostly resembles the *holo* form of LDH. The stiffness of such a region might explain the reduced activity of the thermophilic protein at ambient temperature. However, the observed behavior of the loop does not depend on temperature on the explored time scale. The key question is then, how the loop behaves at the working temperature of the 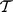 MalDH. According to the *corresponding states* view, at high temperature one would expect the loop to acquire the necessary flexibility to facilitate the binding process. Clearly, a precise characterization of this gating requires to evaluate the kinetic barrier separating the open and close states as well as to evaluate the temperature effect on the transition path. It is possible that the temperature dependence of the atomistic force fields, known to overstabilize proteins, may have influenced the observed response to the temperature excitation. At variance with other investigations using very high temperature stress, we have decided to explore the protein behavior at high but still physical temperature without introducing biases due to unphysical solvent interactions or changes in kinetic paths [56]. Therefore advanced sampling techniques are needed to further investigate the activation of the loop motion. Moreover, it is also possible that the opening of the loop requires a cooperative role from the coenzyme, whose charged groups could trade the stability of the IP network that anchors down this region for an optimized co-enzyme substrate.

## Supporting Information

Figure S1 **Overlap of the thermophilic (orange) and the mesophilic (green) malate dehydrogenases**. PDB codes 4CL3 and 1GV1, respectively.
(TIFF)

Figure S2
**Root mean square deviation of C** ***_α_*** **atoms at** 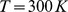 **and** 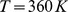 **.** (a) and (b) correspond to the two tetrameric MDH homologues while (c) and (d) show the respective timelines for the monomers simulated in an isolated form.(TIFF)

Figure S3 **Temperature dependence of RMSF.** Derivative of the average RMSF w.r.t. temperature.
(TIFF)

Figure S4 **Hydrogen bonds at the interface.** Number of interdomain protein-protein hydrogen bonds at the 3 different interfaces of the tetramers.
(TIFF)

Figure S5
**Ionic interactions at the** ***d*** **and** ***c*** **interfaces.** (Top panel) Molecular representations of the charged residues between all domains. (Middle panel) Network representation of interfacial IPs for 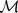 (left) and 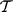 (right) at 300 K. For clarity, only *d* and *c* interfaces are shown as this is where the difference between the two homologues concentrates. The nodes represent charged a.a. that form IPs between different domains. The node-size is proportional to the time the a.a. formed a IPs with any other a.a. The links between the nodes represent salt-bridge formation with thickness proportional to the time the salt-bridge was formed (the largest size is equal 100% of the time). The coloring code refers to the four different domains, green for domain A, blue for B, magenta for C and yellow for D. (Bottom panel) Network of IPs as above but for 360 K. For both temperatures, we can appreciate the high degree of inter-domain connectivity that gives rise to large ion-pair networks in the 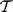 protein (dashed lines). Such a networking is absent in 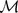. The high degree of connectivity and its dynamical behavior is proposed – yet to be verified with *ad hoc* investigations – to be the source of domain communication during the functional cycle. We also stress that key single-point mutations have been carried out experimentally at the level of residues E25 [53] and E165 [54] in the 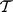 protein. In the former case, at physiological pH, the disruption of the ion-pair connectivity didn't have an important effect on the stability of the tetramer, while when E165 was mutated to either Q or K stability was increased by 24 K.(TIFF)

Figure S6 **Molecular representation of the stiff regions of the two homologous mesophilic (left) and thermophilic (right) pairs.** (Top panel) Our two malate dehydrogenases (MDH) under-study with an explicit colored representation of the residues that get mostly stiffened upon oligomerization. Identification of these residues was done as mentioned in the SI text (see also Fig. S7). For clarity, the residues are shown only for chain A and drawn in three different colors depending on their type. (Bottom panel) Two homologous lactate dehydrogenases (LDH) (PDB codes 2V6B and 2V7P.) The respective stiffened regions are also shown after structural overlap of the two LDH on the two MDH.
(TIFF)

Figure S7
**Relative change of RMSF upon oligomerization at ambient T.** Relative difference between RMSF of chain A in the monomeric and tetrameric form for the mesophilic (left) and thermophilic (right) MDH. Since the quantity plotted is the 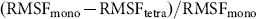, the higher the change the more enhanced the stiffening as we move from the isolated monomer to the tetramer. The residues with RMSF-change larger than 30% (dotted line) were individuated and are shown in color in Fig. S6. Note that for larger thresholds as well as slightly smaller ones, the number of stiff residues for 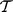 is always larger than for 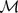.(TIFF)

Table S1 **Electrostatics and hydrophobicity at the interface.** Complementary of Table 3 of the main text in Å^2^. Errors correspond to standard deviation.
(PDF)

Table S2 **Intra- and inter-domain electrostatic energy in kcal/mol at 300K.** Errors correspond to standard deviation.
(PDF)

Text S1 (PDF)
