# Supplementary figures and images for: Interface Matters: The Stiffness Route to Stability of a Thermophilic Tetrameric Malate Dehydrogenase
Source: PLoS One. 2014 Dec 1;9(12):e113895. doi: 10.1371/journal.pone.0113895 (PMC4250060; doi:10.1371/journal.pone.0113895)

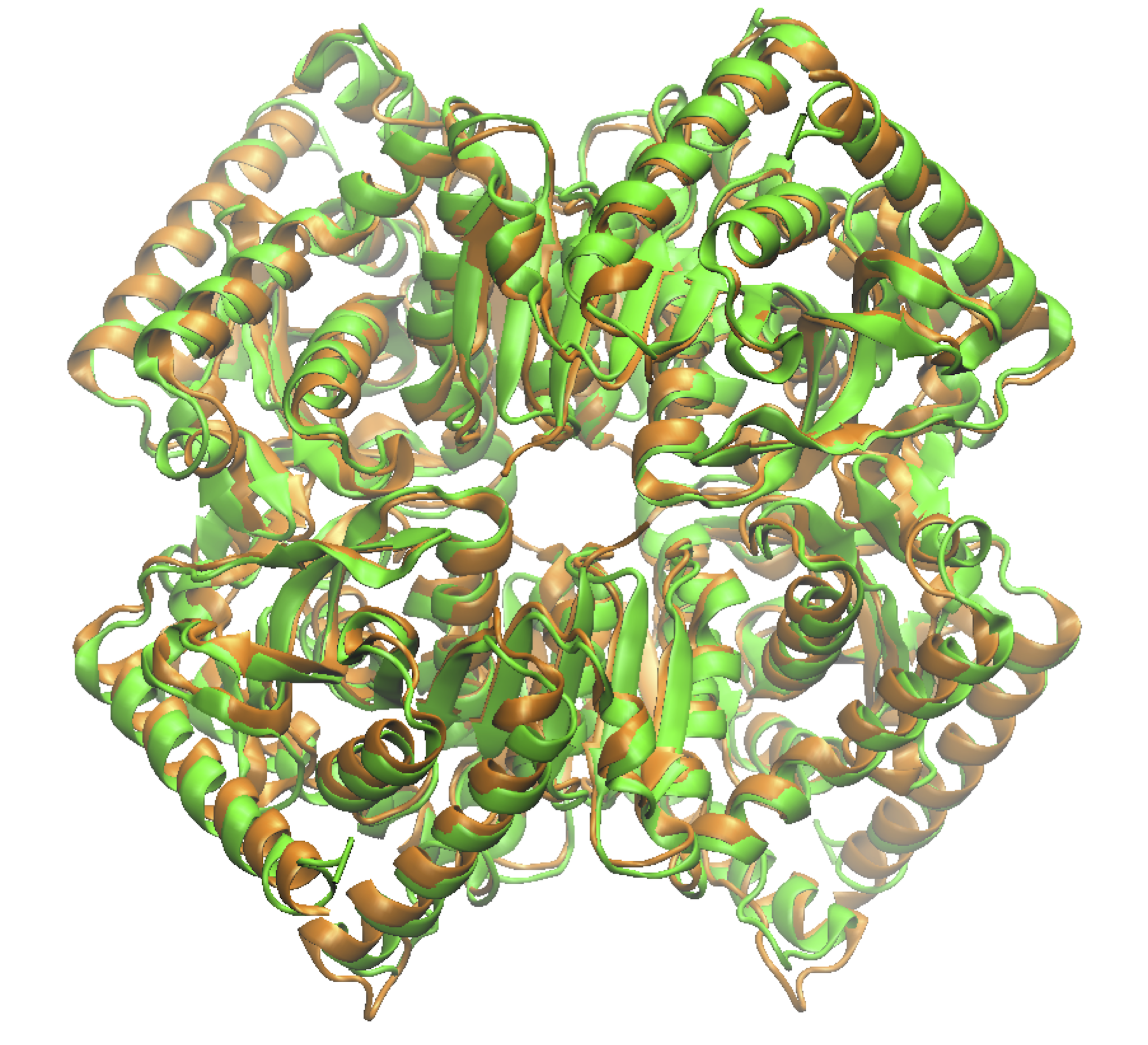

Supplement: Figure S1 — Overlap of the thermophilic (orange) and the mesophilic (green) malate dehydrogenases. PDB codes 4CL3 and 1GV1, respectively. (TIFF) [file pone.0113895.s001.tiff]

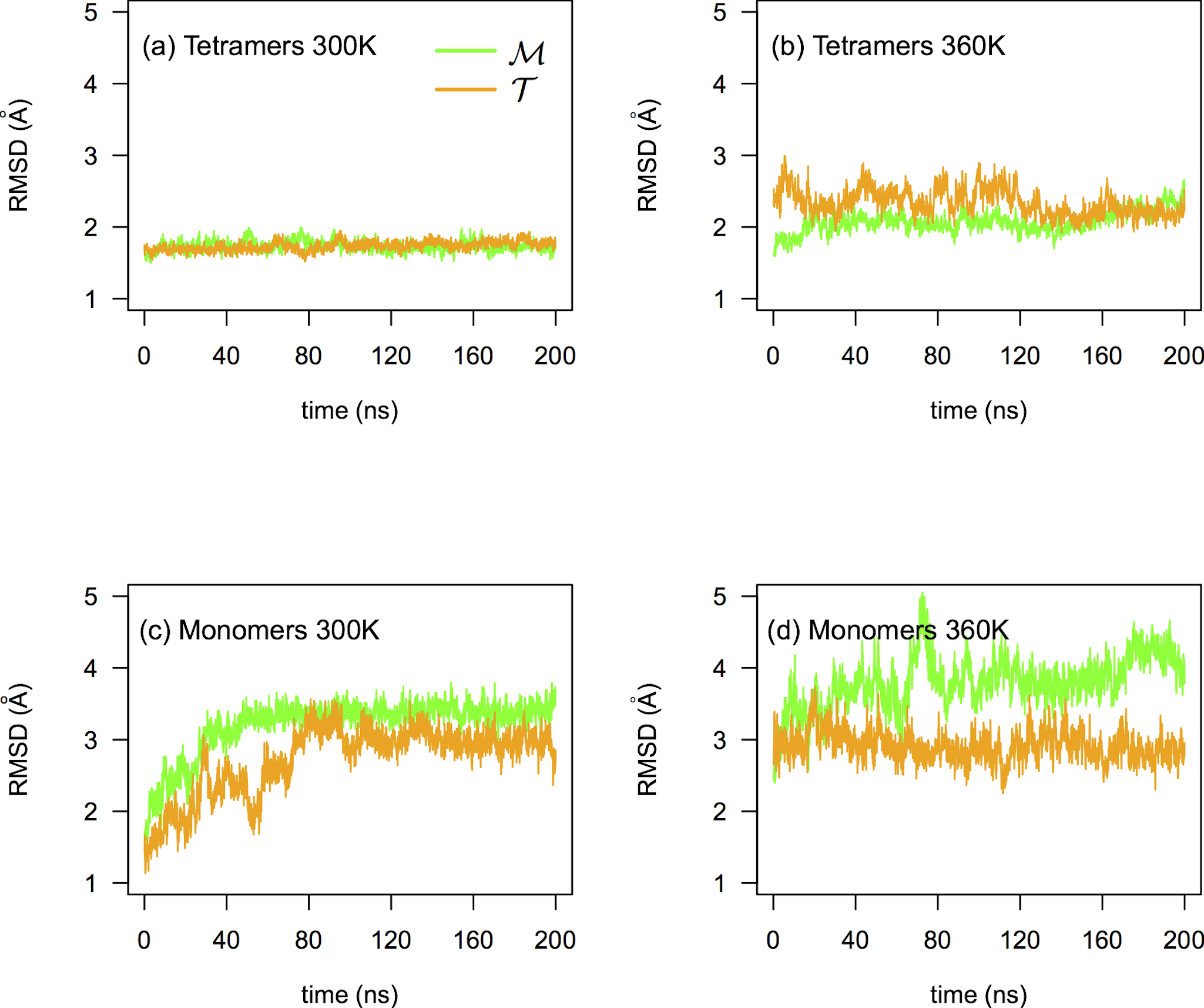

Supplement: Figure S2 — Root mean square deviation of C α atoms at and . (a) and (b) correspond to the two tetrameric MDH homologues while (c) and (d) show the respective timelines for the monomers simulated in an isolated form. (TIFF) [file pone.0113895.s002.tiff]

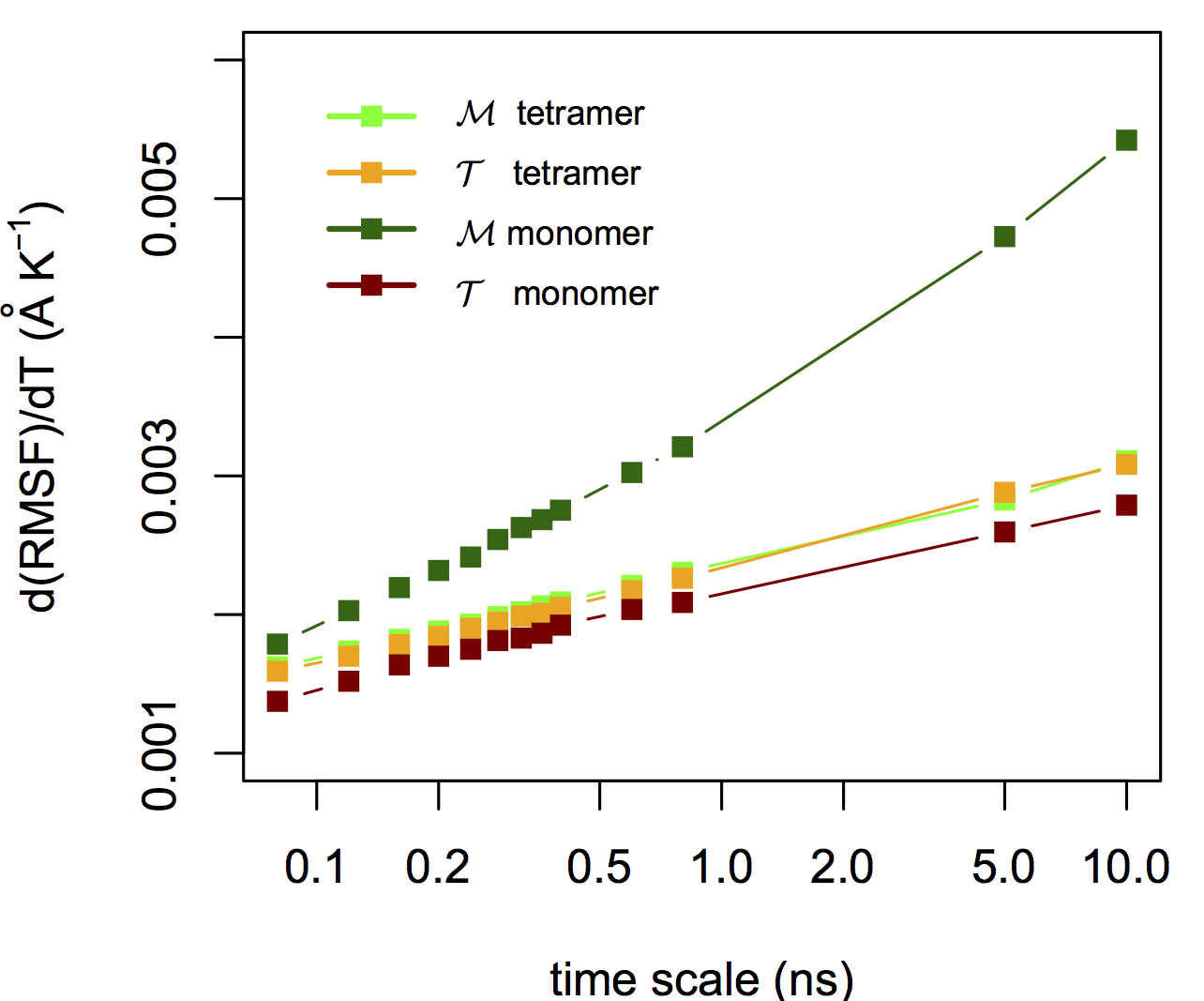

Supplement: Figure S3 — Temperature dependence of RMSF. Derivative of the average RMSF w.r.t. temperature. (TIFF) [file pone.0113895.s003.tiff]

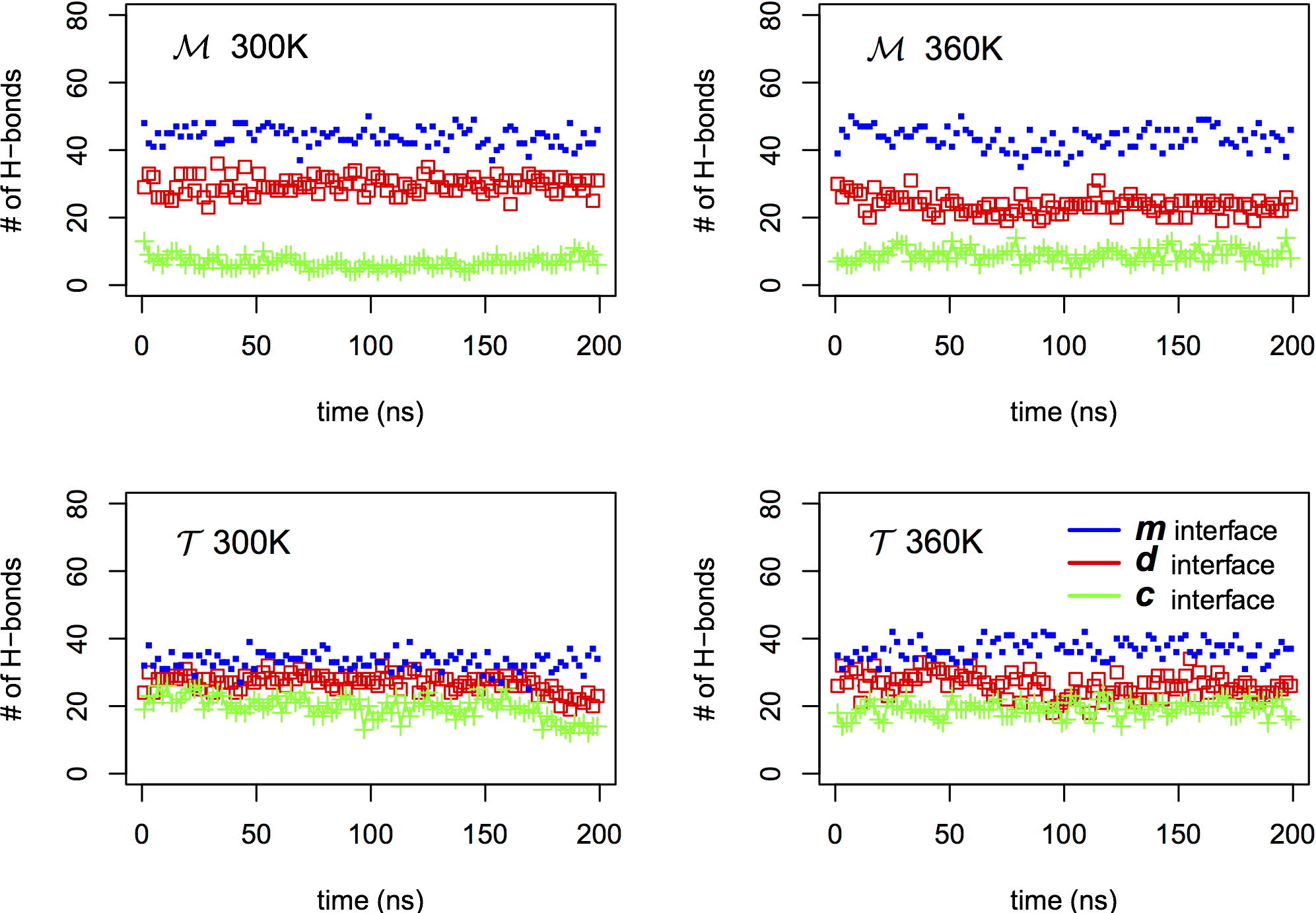

Supplement: Figure S4 — Hydrogen bonds at the interface. Number of interdomain protein-protein hydrogen bonds at the 3 different interfaces of the tetramers. (TIFF) [file pone.0113895.s004.tiff]

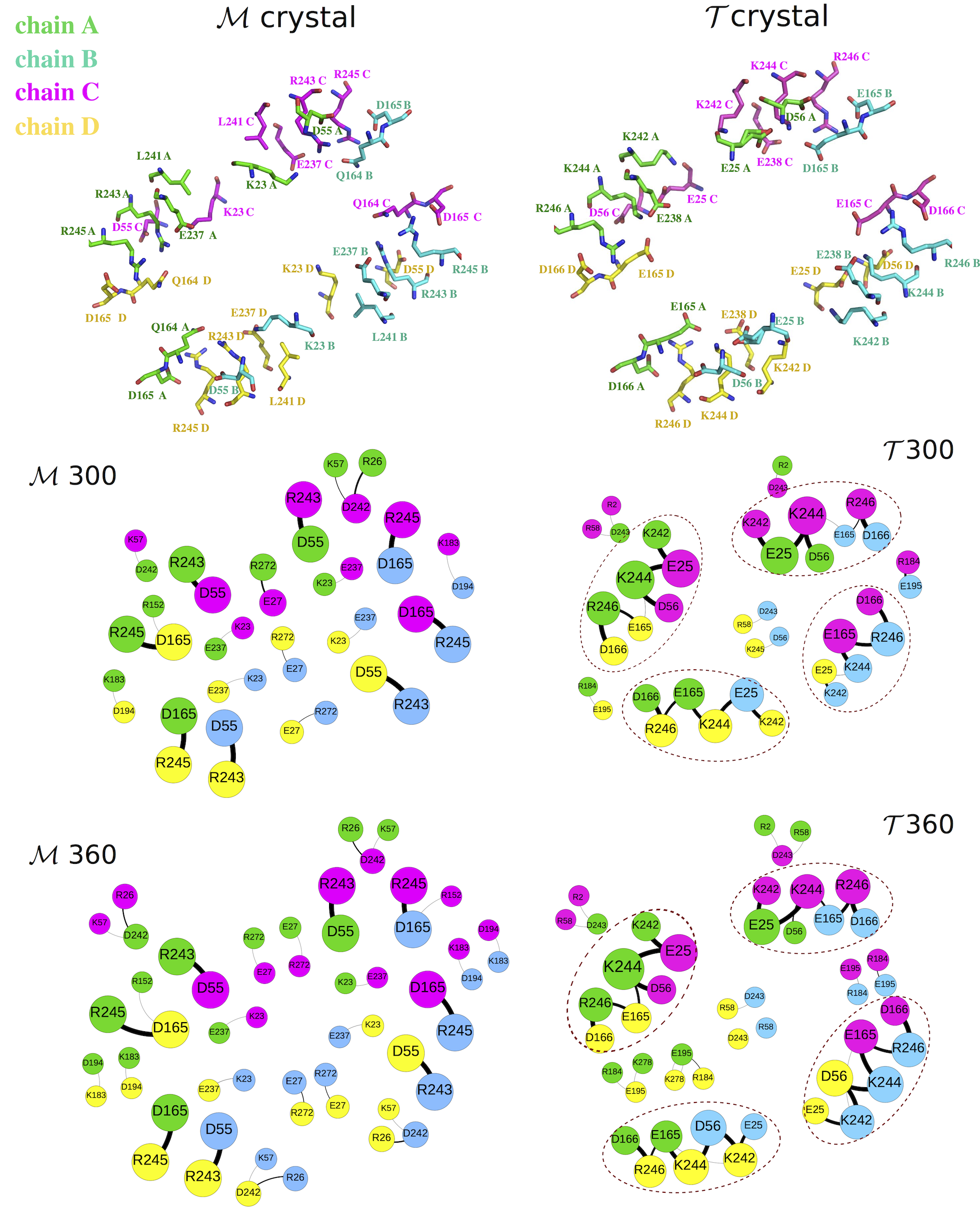

Supplement: Figure S5 — Ionic interactions at the d and c interfaces. (Top panel) Molecular representations of the charged residues between all domains. (Middle panel) Network representation of interfacial IPs for (left) and (right) at 300 K. For clarity, only d and c interfaces are shown as this is where the difference between the two homologues concentrates. The nodes represent charged a.a. that form IPs between different domains. The node-size is proportional to the time the a.a. formed a IPs with any other a.a. The links between the nodes represent salt-bridge formation with thickness proportional to the time the salt-bridge was formed (the largest size is equal 100% of the time). The coloring code refers to the four different domains, green for domain A, blue for B, magenta for C and yellow for D. (Bottom panel) Network of IPs as above but for 360 K. For both temperatures, we can appreciate the high degree of inter-domain connectivity that gives rise to large ion-pair networks in the protein (dashed lines). Such a networking is absent in . The high degree of connectivity and its dynamical behavior is proposed – yet to be verified with ad hoc investigations – to be the source of domain communication during the functional cycle. We also stress that key single-point mutations have been carried out experimentally at the level of residues E25 [53] and E165 [54] in the protein. In the former case, at physiological pH, the disruption of the ion-pair connectivity didn't have an important effect on the stability of the tetramer, while when E165 was mutated to either Q or K stability was increased by 24 K. (TIFF) [file pone.0113895.s005.tiff]

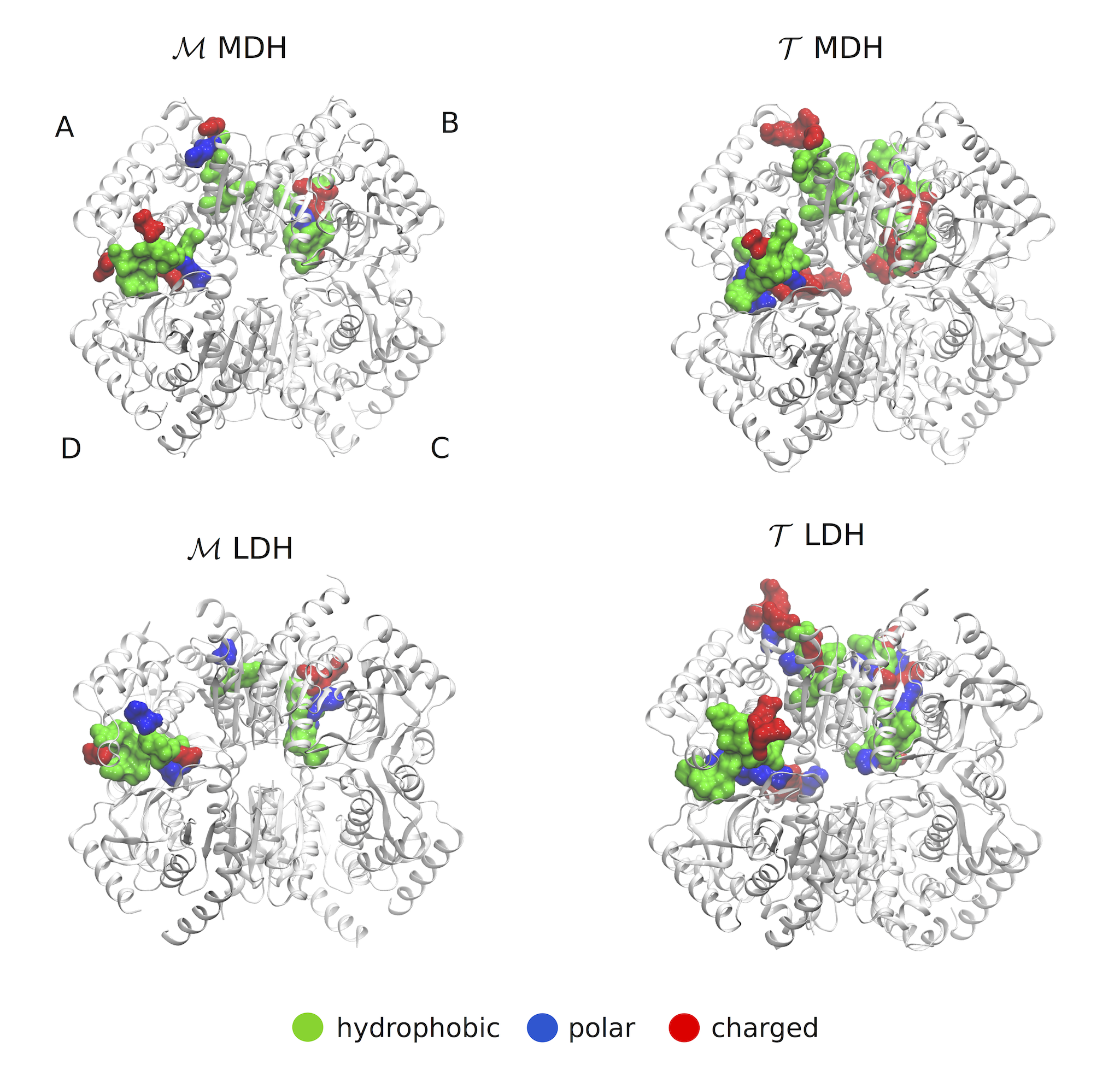

Supplement: Figure S6 — Molecular representation of the stiff regions of the two homologous mesophilic (left) and thermophilic (right) pairs. (Top panel) Our two malate dehydrogenases (MDH) under-study with an explicit colored representation of the residues that get mostly stiffened upon oligomerization. Identification of these residues was done as mentioned in the SI text (see also Fig. S7). For clarity, the residues are shown only for chain A and drawn in three different colors depending on their type. (Bottom panel) Two homologous lactate dehydrogenases (LDH) (PDB codes 2V6B and 2V7P.) The respective stiffened regions are also shown after structural overlap of the two LDH on the two MDH. (TIFF) [file pone.0113895.s006.tiff]

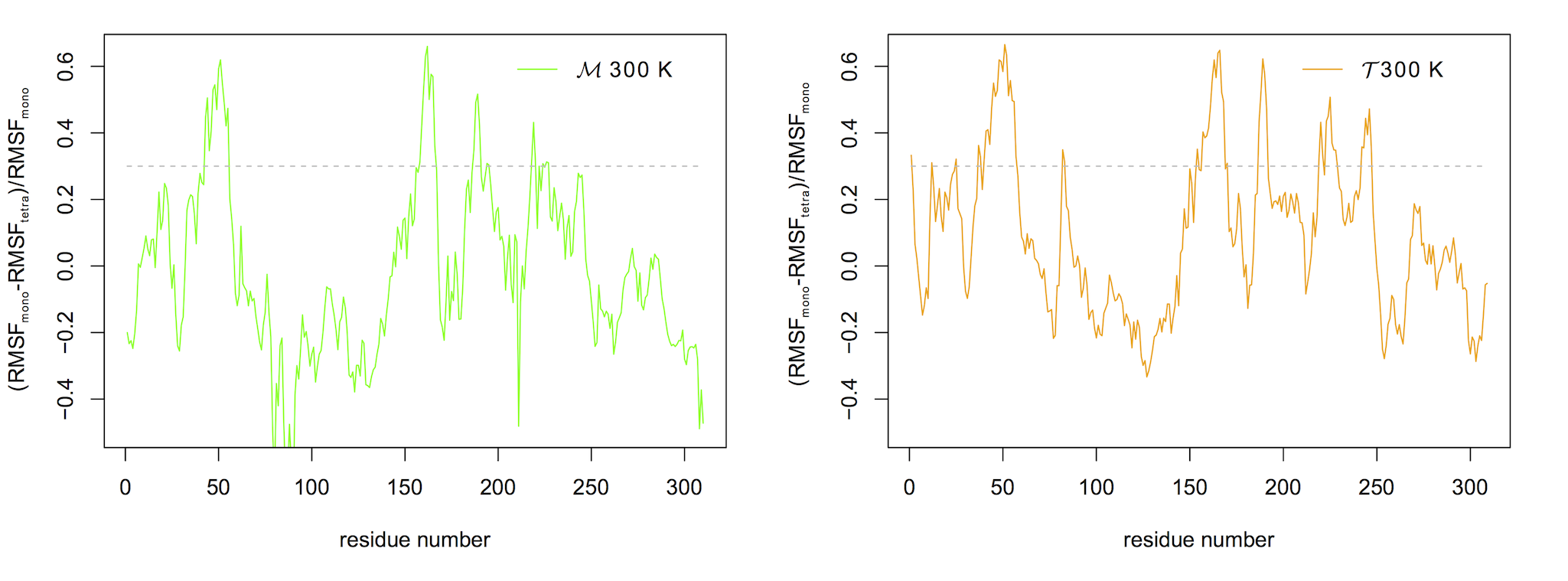

Supplement: Figure S7 — Relative change of RMSF upon oligomerization at ambient T. Relative difference between RMSF of chain A in the monomeric and tetrameric form for the mesophilic (left) and thermophilic (right) MDH. Since the quantity plotted is the , the higher the change the more enhanced the stiffening as we move from the isolated monomer to the tetramer. The residues with RMSF-change larger than 30% (dotted line) were individuated and are shown in color in Fig. S6. Note that for larger thresholds as well as slightly smaller ones, the number of stiff residues for is always larger than for . (TIFF) [file pone.0113895.s007.tiff]
