# Supplementary material for: Interface Matters: The Stiffness Route to Stability of a Thermophilic Tetrameric Malate Dehydrogenase
Source: PLoS One. 2014 Dec 1;9(12):e113895. doi: 10.1371/journal.pone.0113895 (PMC4250060; doi:10.1371/journal.pone.0113895)
Supplement: Table S1 — Electrostatics and hydrophobicity at the interface. Complementary of Table 3 of the main text in Å2. Errors correspond to standard deviation. (PDF) [file pone.0113895.s008.pdf]

Table S1. Electrostatics and hydrophobicity at the interface. Complementary of Table 3 of the main text in absolute values in Å^2

		T = 300 K				
	System	$S_{\text{hydrophobic}}$ (%)	$S_{\text{hydrophilic}}$ (%)	S_{mixed} (%)	H-bonds	N_{IP}
$\mathcal{M}$	*m* interface	629 ± 37	780 ± 31	1391 ± 43	44± 3	9.0 ± 1.2
	d interface	431 ± 27	683 ± 25	384 ± 22	29± 2	4.0 ± 0.2
	c interface	50 ± 6	155 ± 19	168 ± 15	7± 2	1.4 ± 0.7
$\mathcal{T}$	*m* interface	724 ± 29	644 ± 28	1340 ± 39	33± 3	7.5 ± 0.9
	d interface	577 ± 25	634 ± 27	458 ± 29	27± 3	5.9 ± 1.0
	c interface	63 ± 30	223 ± 40	140 ± 43	20± 3	7.8 ± 1.0
		T = 360 K				
$\mathcal{M}$	*m* interface	744 ± 41	825 ± 42	1378 ± 50	44 ± 3	7.3 ± 1.0
	d interface	462 ± 32	609 ± 38	416 ± 28	24 ± 3	4.0 ± 0.3
	c interface	65 ± 10	193 ± 21	163 ± 22	9 ± 2	3.5 ± 0.9
$\mathcal{T}$	*m* interface	782 ± 34	675 ± 36	1285 ± 47	37 ± 3	9.2 ± 0.9
	d interface	531 ± 45	606 ± 49	481 ± 34	27 ± 3	7.3 ± 1.0
	c interface	38 ± 22	198 ± 23	122 ± 37	19 ± 2	9.6 ± 1.2

Errors correspond to standard deviation.
