# Supplementary material for: Interface Matters: The Stiffness Route to Stability of a Thermophilic Tetrameric Malate Dehydrogenase
Source: PLoS One. 2014 Dec 1;9(12):e113895. doi: 10.1371/journal.pone.0113895 (PMC4250060; doi:10.1371/journal.pone.0113895)
Supplement: Table S2 — Intra- and inter-domain electrostatic energy in kcal/mol at 300K. Errors correspond to standard deviation. (PDF) [file pone.0113895.s009.pdf]

Table S2. Intra and inter domain electrostatic energy in kcal/mol at 300K

System		internal	*m* interface	*d* interface	*c* interface
tetra	$\mathcal{M}$	-5550 ± 165	-732 ± 56	-282 ± 36	-155 ± 31
	$\mathcal{T}$	-5214 ± 124	-820 ± 72	-346 ± 55	-512 ± 171
mono	$\mathcal{M}$	-5610 ± 127	-	-	-
	$\mathcal{T}$	-5319 ± 81	-	-	-

Errors correspond to standard deviation.
