# Supplementary material for: Interface Matters: The Stiffness Route to Stability of a Thermophilic Tetrameric Malate Dehydrogenase
Source: PLoS One. 2014 Dec 1;9(12):e113895. doi: 10.1371/journal.pone.0113895 (PMC4250060; doi:10.1371/journal.pone.0113895)
Supplement: Text S1 — (PDF) [file pone.0113895.s010.pdf]

Supporting Information
Interface matters: The stiffness route to stability of a thermophilic tetrameric malate dehydrogenase

Maria Kalimeri[1], Eric Girard[2,3,4], Dominique Madern[2,3,4,*] Fabio Sterpone[1,*]
1 Laboratoire de Biochimie Théorique, Institut de Biologie Physico-Chimique, Centre National de la Recherche Scientifique, UPR9080, Univ. Paris Diderot, Sorbonne Paris Cité, Paris, France
2 Univ. Grenoble Alpes, Institut de Biologie Structurale, Grenoble, France
3 Centre National de la Recherche Scientifique, Institut de Biologie Structurale, Grenoble, France
4 Commissariat à l'Energie Atomique et aux énergies alternatives, Institut de Biologie Structurale, Grenoble, France
∗ E-mail: Corresponding authors fabio.sterpone@ibpc.fr, dominique.madern@ibs.fr

Stiffening upon oligomerization

The top panel of Fig. S6 shows, in color, the molecular representations of the mesophilic (left) and thermophilic (right) residues whose RMSF is more than 30% decreased upon oligomerization. These residues are individuated as those having the quantity $(\mathrm{RMSF}_{\mathrm{mono}} - \mathrm{RMSF}_{\mathrm{tetra}})/\mathrm{RMSF}_{\mathrm{mono}}$ larger than 0.3 (see Fig. S7). For this purpose the RMSF was calculated in rather long windows of 10 ns and averaged over the total trajectory length (200 ns). This was because larger timescales had obviously a more pronounced effect in RMSF decrease. Strikingly, the number of stiffened residues for $\mathcal{T}$ is much larger than for $\mathcal{M}$ (59 versus 34, respectively). Even more so, as can be seen in red in the top panel of Fig. S6, among those residues 29% are charged for $\mathcal{T}$ versus 21% for $\mathcal{M}$ (also 12% polar and 59% hydrophobic for $\mathcal{T}$ versus 12% polar and 67% hydrophobic for $\mathcal{M}$). Additionally, the vast majority of those charged residues forms ion-pairs that survive during the total length of the simulation for both $\mathcal{M}$ and $\mathcal{T}$. Thus for this pair of homologous proteins we see a clear correlation between electrostatic interactions and rigidity. For the sake of comparison, we overlapped the structure of $\mathcal{M}$ MDH on that of a homologous mesophilic LDH (PDB code 2V6B) and the structure of $\mathcal{T}$ MDH on that of a homologous thermophilic LDH (PDB code 2V7P) and individuated the equivalent set of residues. Analogously, 23% of them are charged for $\mathcal{T}$ LDH versus 15% for $\mathcal{M}$ LDH. Those regions can be seen in the bottom panel of Fig. S6. For the latter result, the obvious assumption is that the rigid regions between MDH and LDH are the same.
